# MicroRNA-466 with tumor markers for cervical cancer screening

**DOI:** 10.18632/oncotarget.19992

**Published:** 2017-08-07

**Authors:** Li-Li Zhou, Yong Shen, Jiao-Mei Gong, Ping Sun, Jia-He Sheng

**Affiliations:** ^1^ Department of Clinical Laboratory, Affiliated Cancer Hospital of Zhengzhou University & Henan Cancer Hospital, Zhengzhou, Henan, China; ^2^ Department of Clinical Laboratory, Second Affiliated Hospital of Zhengzhou University, Zhengzhou, Henan, China

**Keywords:** microRNA-466, cervical cancer, tumor marker, screening, diagnoses

## Abstract

Cervical cancer is the second most common cancer in women in the world. In this study, we explore tumor markers and microRNA-466 combination for cervical cancer screening. Tumor markers were measured by the methods of electro-chemiluminescent immunoassay and enzyme immunoassay. The microRNA-466 was performed by quantitative real-time polymerase chain reaction. Among normal group, hyperplasia group and cancer group, the CEA expression levels were 2.26 ng/ml, 3.85 ng/ml and 16.08 ng/ml, respectively. While the CA125 expression levels were 13.61 u/ml, 27.32 u/ml and 44.93 u/ml, respectively. The SCCA expression levels were 13.61 ng/ml, 27.32 ng/ml and 44.93 ng/ml, respectively. The expression levels of tumor markers were all gradually increased with the development of cervical lesions. The expression levels of microRNA-466 in cervical cancers (0.62) were greater than that in normal (0.076) and hyperplasia (0.24). The expression of microRNA-466 was correlated with lymphnode metastasis (*P*=0.000). There is a lower overall survival rate of patient with large tumor or lymphnode metastasis. Thus, the combination of tumor markers and microRNA-466 can be useful for early detection of cervical cancer and indicators for advanced stage and prognosis of the disease.

## INTRODUCTION

Cervical cancer is the second most frequent cancer in women and the most common gynecological cancer [1]. In 2008, there were about 529,800 cases included 275,000 deaths worldwide due to cervical cancer [2]. Moreover, 85% of new cases occur in developing countries where the survival rates are substantially lower as a result of a presentation at relatively advanced stages [3]. This condition affects severely the health and lives with the women [4].

MicroRNAs (miRNAs), small non-coding short RNAs of 18-25 nucleotides in length, generally act as negative regulators of gene expression at the post-transcriptional level through mRNA degradation or translation repression [5, 6]. These molecules, as key regulators of cellular growth and differentiation, play an important role in many biological processes [7, 8]. MicroRNAs were generally down-regulated or up-regulated in malignant tissues [9, 10]. The expression levels of MicroRNA-466 in cervical cancer tissue have been reported in our previous study [11].

Tumor markers, which are small-molecule matters produced by tumor cells or generated by host cells in response to the tumor, are frequently used for screening and monitoring in oncology [12, 13]. However, the same tumors can express several different unique antigens [13, 14]. Recently, normal cervical cells have been reported to synthesize and secrete the tumor marker carcinoembryonic antigen (CEA) as a frequent constituent of normal cervical mucus [15]. The cancer antigen 125 (CA125), a transmembranes glycoprotein, is a tumor marker mainly utilized for the diagnosis and treatment of epithelial ovarian cancer and cervical cancer [16]. Squamous cell carcinoma antigen (SCCA) is separated from a squamous cell carcinoma (SCC) of the uterine cervix [13]. These results suggest that cervical adenocarcinoma may excrete elevated levels of CEA and/or SCCA in cervical cancer instead of CA125. Therefore, we measured tumor markers (CEA, CA125, and SCCA) and MicroRNA-466 in the serum levels from women with or without cervical cancer, and analyzed the results to clarify whether it helps in the early diagnosis of cervical cancer or not.

## RESULTS

### The expression levels of tumor markers and microRNA-466

**Figure 1:**
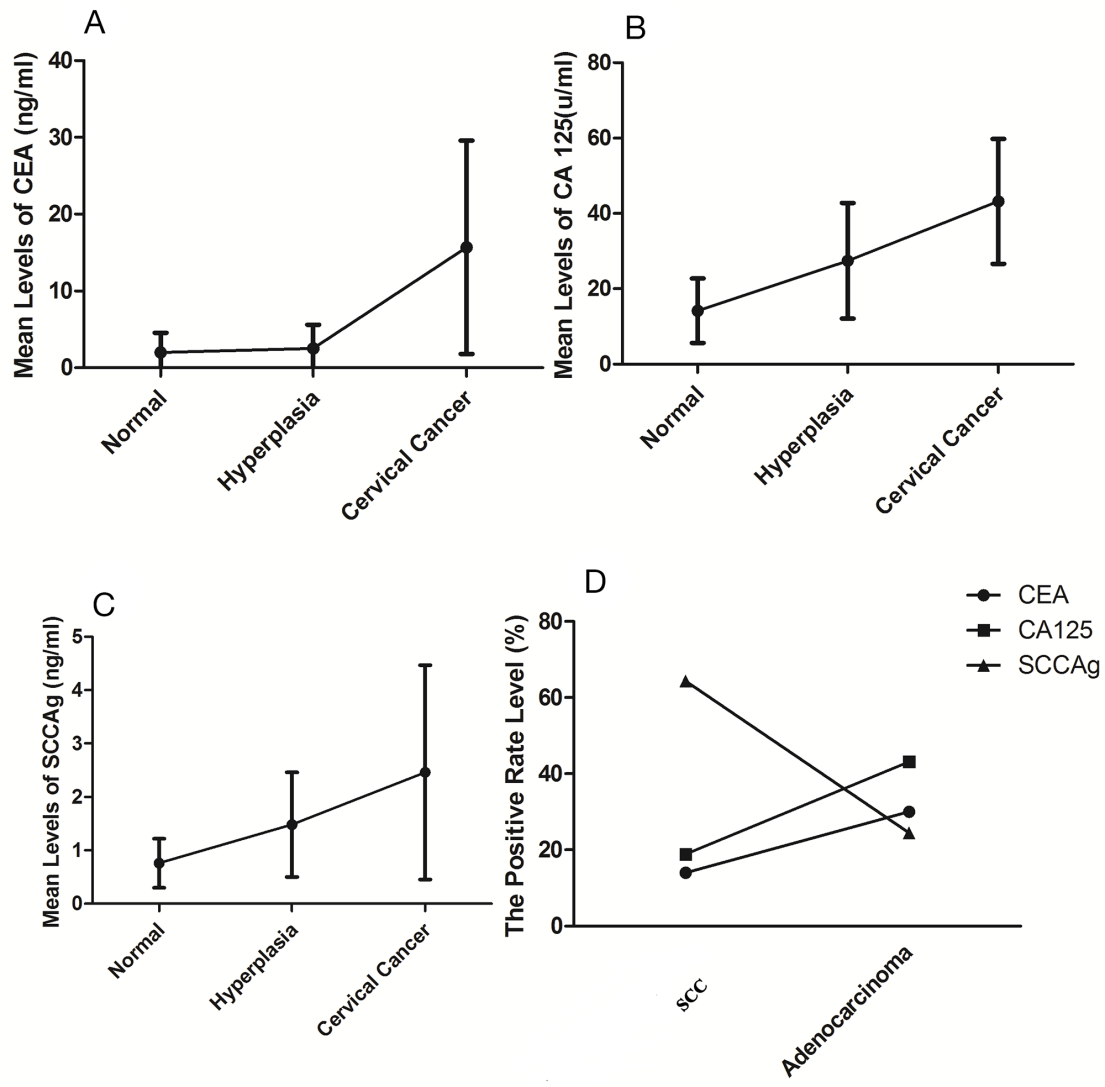
The expression levels of tumor markers **(A)** Mean expression levels of CEA in the three groups. **(B)** Mean expression levels of CA125 in the three groups. **(C)** Mean expression levels of SCCA in the three groups. **(D)** The positive rate level of tumor markers between SCC and cervical adenocarcinoma.

A total of 735 patients were enrolled in this study, including 245 cervical cancer patients, 280 cervical hyperplasia patients and 210 normal people. The expression levels of CEA in the normal group, hyperplasia group and cancer group were 2.26 ng/ml, 3.85 ng/ml and 16.08 ng/ml, respectively (Figure 1A). It was shown that the expression levels of CEA were gradually built up over the development of cervical lesions. The expression levels of CA125 in three groups were 13.61 u/ml, 27.32 u/ml and 44.93 u/ml, respectively (Figure 1B). Similarly, expression levels of SCCA were 13.61 ng/ml, 27.32 ng/ml and 44.93 ng/ml, respectively (Figure 1C). The increased trend of CA125 and SCCA was similar with CEA.

**Figure 2:**
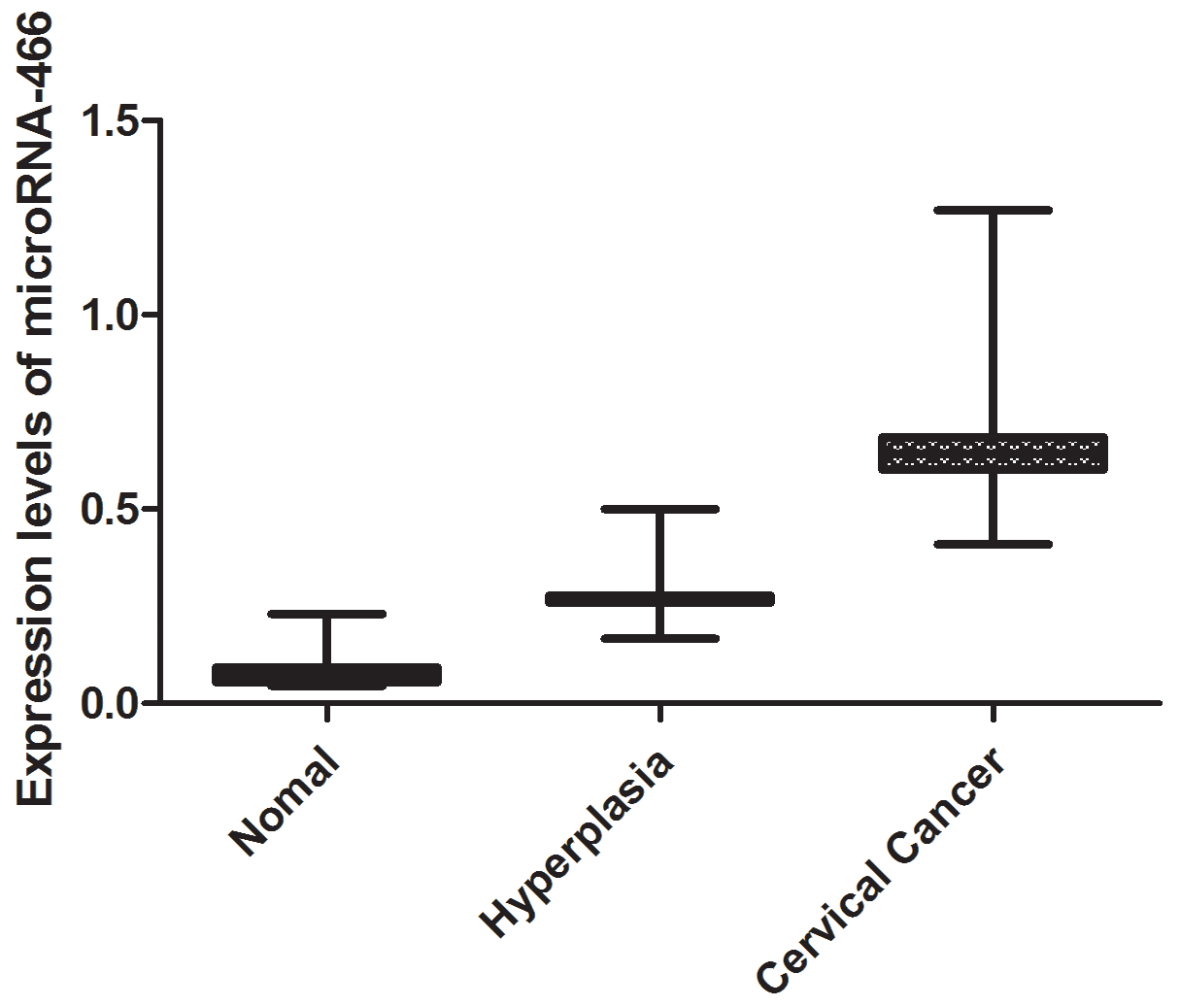
The expression levels of microRNA-466

We tested serum expression levels of microRNA-466 by quantitative real-time PCR in cervical cancer group, hyperplasia group and normal group. It can be seen that significant differences existed in the MicroRNA-466 expression level from the three groups (Figure 2). The averages fold changes of MicroRNA-466 in patients with cervical cancer group and normal group were 0.62 and 0.076, respectively (*P*<0.01), and that in patients with hyperplasia group and people group was 0.24 and 0.076, respectively (*P*<0.01).

### The expression levels of microRNA-466 in cervical cancer patients

There were 245 cervical cancer patients, including 143 squamous cell carcinoma (SCC) patients and 102 adenocarcinomas (AC) patients. Cervical cancer patients’ characteristics were described in Table 1. There was no significant difference in age, the international federation of gynecology and obstetrics (FIGO), and tumor size between the SCC patients and AC patients (*P*=0.076). However, AC was more prone to lymph node metastasis than SCC (*P*=0.039). Interestingly, microRNA-466 expression was likewise higher in the lymph node metastatic than in the negative lymph node (*P*=0.000).

**Table 1:** The primer sequences of real-time PCR for microRNA-466

	Primer	Sequences	Product
miR-466	RT stem-loop primer	5’-GTCGTATCCAGTGCAGGGTCCGAGGTATTCGCACTGGATACGACATGTGTGT-3’	57
	Forward primer	5’-ATGGTTCGTGGGATACACATACACGCA-3’	
	Reverse primer	5’-GCAGGGTCCGAGGTATTC-3’	
U6	RT primer	5’-CGCTTCACGAATTTGCGTGTCAT-3’	101
	Forward primer	5’-GCTTCGGCAGCACATATACTAAAAT-3’	
	Reverse primer	5’-CGCTTCACGAATTTGCGTGTCAT-3’	

SCCA was raised in 63.8% of SCC patients at diagnosis (Figure 1D). Levels of CEA and CA125 in SCC patients were elevated in 14.3% and 18.9%, respectively. However, CA125 was preeminent in 43.1% of AC patients at diagnosis. Levels of CEA and SCCA of AC patients were only elevated in 29.2% and 21.6%, respectively.

### Overall survival curves for cervical cancer

**Figure 3:**
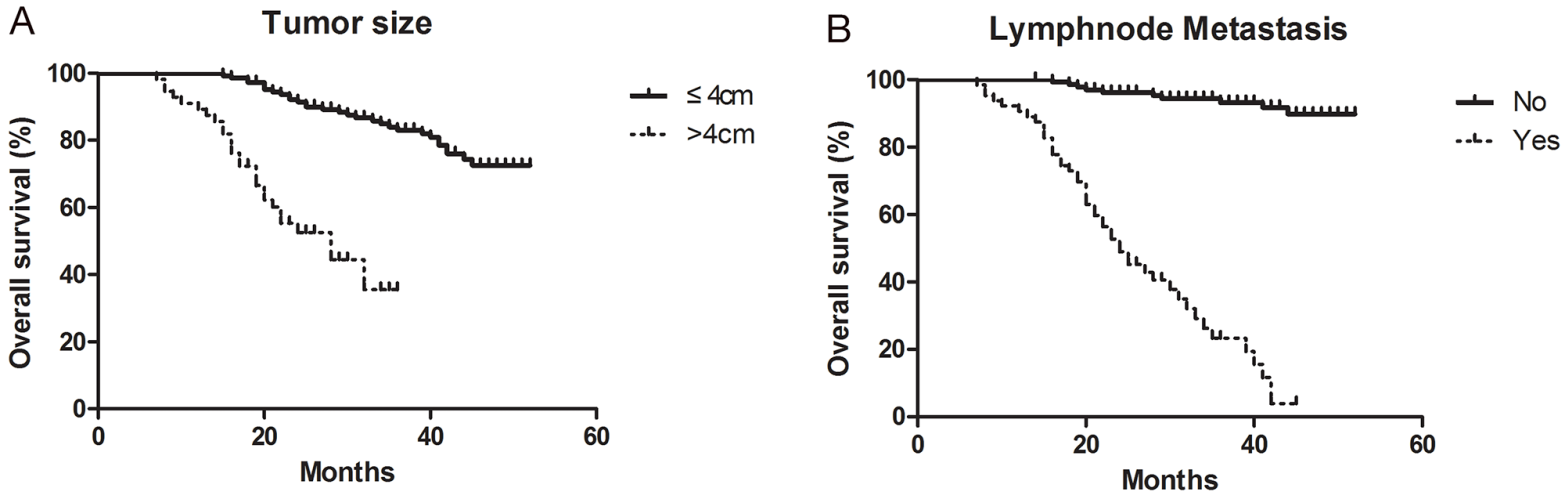
Overall survival curves for cervical cancer **(A)** The overall survival rate of patient between small tumor (≤4cm) and large tumor (>4cm). **(B)** The overall survival rate of patient with or without lymphnode metastasis.

The median overall survival was 32.6 months (95% CI, 7 months to not estimable). Compared to the patient with small tumor (≤4cm), there is a low overall survival rate of patient with large tumor (>4cm) (Figure 3A). The overall survival rate of patient with lymphnode metastasis is lower than patient without lymphnode metastasis (Figure 3B). There was a significant difference.

### AUC for diagnosing cervical cancer

It showed the AUC values of preoperative tumor markers and microRNA-466 for predicting high-risk factors in the training set (Table 2). The estimated logistic regression model on receiver operating curves (ROC) analysis was utilized. We used c-statistics to calculate the AUC of the ROC. The AUC of the combination was shown in Table 3. The combination of tumor markers and microRNA-466 can be a better diagnosis of cervical cancer (AUC=0.85).

**Table 2:** Clinical characteristics of 245 cervical cancer patients

Characteristics	SCC(%) n=143	AC(%) n=102	*P*^a^ Value	Expression of MiR-466 (M±SEM)	*P*^a^ Value
Age (years)					
≤50	59 (42.3)	45 (44.1)	0.695	0.616±0.151	0.733
>50	84 (58.7)	57 (55.9)		0.623±0.164	
FIGO stage					
I, IIa	92 (64.3)	68 (66.7)	0.706	0.612±0.147	0.219
IIb, III-IV	51 (35.7)	34 (33.3)		0.635±0.123	
Tumor size					
≤4cm	104 (72.7)	70 (68.6)	0.486	0.609±0.160	0.076
>4cm	39 (27.3)	32 (31.4)		0.647±0.128	
Lymph node					
Negative	106 (74.1)	63 (61.8)	0.039	0.582±0.197	0.000
Positive	37 (25.9)	39 (38.2)		0.705±0.178	

SCC: squamous cell carcinoma; AC: adenocarcinoma;

FIGO: International Federation of Gynecology and Obstetrics.

^a^ Student’s t test.

**Table 3:** AUC of different combinations for diagnosing cervical cancer

	AUC
Univariate	
CEA	0.55
CA125	0.62
SCCA	0.65
MicroRNA-466	0.48
Multivariate	
CEA+CA125	0.71
CEA+SCCA	0.70
CA125+SCCA	0.75
CEA+CA125+SCCA	0.80
CEA+CA125+SCCA+ MicroRNA-466	0.85

AUC: area under the receiver–operator curve; CEA: carcinoembryonic antigen; CA125: cancer antigen 125; SCCA: squamous cell carcinoma antigen.

## DISCUSSION

Cervical cancer which is the second most common cancer in women has become a major-health care problem throughout the world [1]. The 5-year survival rate is only 38% to 56% for an advanced stage [17]. Therefore, it is important to diagnose cervical cancer at an early stage. Tumor markers have been widespread used in clinical medicine [18, 19]. Tumor markers can offer early diagnosis, prognosis evaluation and therapeutic efficacy evaluation [20].

CEA was first identified in the serum of patients with colonic carcinoma [21]. Several studied to have shown CEA was positivity in an abdominal tumor, including cervical cancer [22, 23]. CA 125, produced by epithelial ovarian tumors, has been put forward as a tumor marker of ovarian cancer [24]. CA125 can be also expressed in cervical glands [16]. Recently, normal cervical glandular cells have been recorded to synthesize and secrete the tumor marker CA 125 into normal cervical mucus [16, 25]. Numerous studies have shown SCCA levels were correlation with the high-risk factors in early stage of SCC [13, 26]. In our study, we showed that the expression levels of CEA, CA125 and SCCA were all gradually increased by the development of cervical lesions. The expression levels of CEA and CA125 were highly expressed in cervical adenocarcinoma. SCCA was relatively specific for cervical squamous carcinoma. Another study showed that elevated SCCA levels were associated with lymph node metastasis among high-risk factors [27]. However, our study showed AC was more prone to lymph node metastasis than SCC (*P*=0.039).

The gene of microRNA-466 is located on the short arm of the third chromosome (3p23). MicroRNA-466, first discovered from melanoma in 2010 [28], has been declared as a marker of lung cancer caused by smoking [29]. Genome sequence analysis and bioinformatics prediction indicate that microRNA-466 is highly homologous with the LCR region prediction miRNA of HPVl6/18, while the LCR region is the regulatory region of carcinogenic key protein expression [30]. Lots of evidence has shown that high-risk type HPV (HPV16/18) infection is a prominent risk factor for cervical cancer development [31]. MiRNAs may act as a bio-marker in cervical cancer tissue or cervical lesions which are related to HPV16/18 infection [32, 33]. The expression of microRNA-466 in cervical cancer group was significantly greater than in normal people group. It was suggested that microRNA-466 might play a part in the pathogenesis cervical cancer progression.

Our study was designed to diagnose cervical cancer at the primary stage. The multivariate analysis of ROC curve was carried out to assess the value of various combinations of three tumor markers for the diagnosis of cervical cancer. Our results showed that the AUC for objective tumor markers was all below 0.7. Using a combination of tumor markers and microRNA-466, the AUC can reach 0.85. One crucial question was why the combination correlates with the diagnosis levels of cervical cancer. Unfortunately, the answer for this question was outside the scope of the present investigation. The biologic functions of tumor markers were largely unknown and must be elucidated to respond to this question in the future [27]. The combination of the three tumor markers can be utilized to screening. On multivariate analysis of the ROC in the present study, it was difficult to ascertain the cut-off level for each tumor marker. Therefore, we used the cut-off levels recommended by the manufacturers of the test kits.

In conclusion, our results indicate that the new combination of serum tumor markers and microRNA-466 is closely associated to the occurrence and development of cervical cancer and can be utilized to screening for cervical cancer.

## MATERIALS AND METHODS

### Samples and clinical data

A total of 735 patients were recruited for this study at Affiliated Cancer Hospital of Zhengzhou University from January 2013 to June 2016. They were divided into three groups by pathology, namely normal group (n=210), hyperplasia group (n=280), and cervical cancer group (n=245). The median age of the 735 patients was 49 years old (ranging from 26 to 72 years old). The selection criteria for patients were as follows: (1) pathologically confirmed patients with cervical cancer, cervical hyperplasia, or normal; (2) the patients had no history of other cancers; (3) No patients had prior treatment history. All the patients were reviewed by microRNA-466 and tumor markers (CEA, CA125 and SCCA) in serum levels. This study was subject to approval by the Ethics Committee of Affiliated Cancer Hospital of Zhengzhou University. All participants had a written informed consent which was consulted on all subjects. We confirmed that all methods were performed in accordance with the applicable guidelines and regulations. All identifying information of the patient had been removed from all text/figures/tables/images.

### Tumor markers analysis

Electro-chemiluminescent immunoassay was used to identify the CEA and CA125 levels in serum using an automatic Roche Cobas E602 analyzer (Roche Diagnostics, Indianapolis, IN). The limit of detection was established by analyzing 10 replicates of the zero calibrators and 4 replicate of the lowest nonzero calibrator. The limit of detection for CEA and CA125 in human serum was determined to be 0.2ng/ml and 0.50 U/ml, respectively. SCCA was monitored by enzyme immunoassay. The cut-off levels recommended by the manufacturers of the test kits were used. That is, 3.5ng/mL for CEA, 35 U/ml for CA125, and 2.2 ng/ml for SCCA.

### RNA extraction and quantitative real-time polymerase chain reaction

The microRNA-466 was performed by quantitative real-time polymerase chain reaction. Total RNA was extracted and isolated from serum by using Trizol Reagent (Shanghai Sangon) according to the manufacturer’s instructions. All the RNA was reverse-transcribed using the PrimeScript® RT reagent Kit (TaKaRa). The PCR conditions were as follows: 37 °C for 15 min, 85 °C for 5 s, and 4 °C for preservation. MiRNA quantification was performed as described for the Applied Biosystems real-time PCR system using stem-loop primers for microRNA-466. RT-qPCR using SYBR® Premix Ex Taq™ II kit (TaKaRa). The PCR amplification conditions were as follows: 94°C denaturation for 2min, 94°C for 15sec, 57°C for 20sec, 72°C for 15sec, 35cycles. The expression level of microRNA-466 was calculated by comparative cycle threshold (Ct) method. U6 was selected as internal control since its expression levels were stable in both patients with cervical cancer and non-cancer controls, and the Ct value of miR-466 was normalized in relation to that of U6. For calculations of fold changes we used the 2-ΔΔCT method. Primer sequences are listed in Table 3. The microRNA-466 cut-off level recommended by normal people was 0.1.

### Statistical analysis

All statistical analyses were performed using SPSS 17.0 statistical packages for Windows (SPSS Inc., Chicago, IL, USA). All values were presented as M (Mean) ± SEM (Standard Error of Mean). The Student’s t-test analysis was carried out to analyze the clinical data. Overall survival curves were used to assess the prognosis of cervical cancer. Areas under receiver operator curves (AUC) were estimated using a logistic regression model. All *P*-values were two-sided, and *P*≤0.05 was deemed to indicate statistical significance.
